# Two Speed Factors of Visual Recognition Independently Correlated with Fluid Intelligence

**DOI:** 10.1371/journal.pone.0097429

**Published:** 2014-05-13

**Authors:** Ryosuke Tachibana, Yuri Namba, Yasuki Noguchi

**Affiliations:** Department of Psychology, Graduate School of Humanities, Kobe University, Nada, Kobe, Japan; University of Maryland, College Park, United States of America

## Abstract

Growing evidence indicates a moderate but significant relationship between processing speed in visuo-cognitive tasks and general intelligence. On the other hand, findings from neuroscience proposed that the primate visual system consists of two major pathways, the ventral pathway for objects recognition and the dorsal pathway for spatial processing and attentive analysis. Previous studies seeking for visuo-cognitive factors of human intelligence indicated a significant correlation between fluid intelligence and the inspection time (IT), an index for a speed of object recognition performed in the ventral pathway. We thus presently examined a possibility that neural processing speed in the dorsal pathway also represented a factor of intelligence. Specifically, we used the mental rotation (MR) task, a popular psychometric measure for mental speed of spatial processing in the dorsal pathway. We found that the speed of MR was significantly correlated with intelligence scores, while it had no correlation with one’s IT (recognition speed of visual objects). Our results support the new possibility that intelligence could be explained by two types of mental speed, one related to object recognition (IT) and another for manipulation of mental images (MR).

## Introduction

Differences among individuals in their mental abilities are important and controversial issues in psychology [1]. The most popular index for those mental abilities is the intelligence quotient (IQ) in various intelligence tests. In standard tests such as the Wechsler Adult Intelligence Scale (WAIS) [2], the IQ is measured as the average performance of a total set of cognitive functions, such as long-term memory, short-term memory, calculation, language, spatial processing, attention, and reasoning. These multifaceted aspects of intelligence, however, sometimes make it unclear what kind of cognitive components or elements actually contribute to one’s mental ability or intelligence.

For a better understanding of IQ and human intelligence, some studies have attempted to find visuo-cognitive bases of intelligence in simple psychometric measures [3], such as accuracy and reaction times in visual tasks. Of particular interest in these days is a close relationship between intelligence and processing speed in visuo-cognitive tasks [4], [5]. This line of studies generally assumes that a brighter person processes information more rapidly and hence has higher mental abilities [6]–[8]. A typical example for this relationship of intelligence with mental speed is an inspection time (IT) [9]. In the IT task, one trial begins with a presentation of a target stimulus, immediately followed by a mask stimulus that prevents the recognition of the target. The IT is defined as a minimal stimulus onset asynchrony (SOA) between the target and mask at which a subject can recognize the target. Previous studies showed a moderate but significant correlation (around *r* = 0.3) between the IT and intelligence measures [10]; a person with a higher intelligence showed a shorter IT and thus has a higher speed of visually presented object recognition.

On the other hand, findings from neuroscience proposed the two-pathway model of visual recognition [11]. This model assumes that the primate visual system comprises two main pathways, the ventral and dorsal pathways. The ventral pathway, projecting from the primary visual cortex (V1) to the inferior temporal cortex, is dedicated to processing object identities [12]. In contrast, the neural processing for spatial information of visual stimuli is mainly performed in the dorsal pathway that projects from the V1 to parietal cortex. The dorsal pathway is also important as a part of cortical networks for the control of attention [13]. Since the IT task involves recognition of the target shape as the backward masking paradigm, this task is thought to be related more closely with the processing in the ventral than dorsal pathway [14].

Those views from neuroscience, being combined with the significant correlation between IT and intelligence, prompt us to seek for another cognitive factor of intelligence that reflects a speed of neural processing in the dorsal (not ventral) pathway. Indeed, a previous study used the attentional blink (AB), a psychological phenomenon closely related to the neural processing in the dorsal pathway [15], [16], and investigated an inter-individual correlation between magnitudes of AB and intelligence scores. They, however, failed to find any relationship between those two, suggesting that a psychophysical measure of attention (an index for the neural processing in the dorsal pathway) is *not* strongly connected with intelligence [17].

In the present study, we thus focused on another function of the dorsal pathway, the spatial processing of visual stimuli. Specifically, we used the mental rotation (MR) task, a typical cognitive test that measures a speed of spatial encoding and manipulation of mental images [18]. Previous studies using fMRI and PET have shown a strong activity in the parietal cortex when subjects performed the MR task [19]–[22]. Another study of event-related potential (ERP) showed that the amplitude of the potential in the parietal cortex correlated negatively with the reaction time (RT) in MR task [23]. By investigating relationships among three cognitive measures (IT, MR, and IQ) on the same set of subjects, we examined a possibility that neural processing speed in the dorsal pathway (as was indexed by the MR task) represented a factor of intelligence.

## Methods

### Subjects

Forty-seven subjects (23 females, age: 19–29) participated in this experiment. They have normal or corrected-to-normal vision. Data of one subject were excluded from analyses because of a low performance and a low determination coefficient of a linear fitting in the MR task (see below). Informed consent was received from each subject after the nature of the study had been explained. Approval for the experiment was obtained from the ethics committee of Kobe University, Japan.

### Stimuli and Tasks

Each subject performed the following three tasks sequentially; the MR, IT, and the Raven’s advanced progressive matrices test (APM). The order of those three tasks was counterbalanced across subjects.

#### Mental Rotation (MR) task

All visual stimuli were generated using Matlab Psychophysics Toolbox [24], [25] and presented on a CRT screen at a refresh rate of 100 Hz. Conforming to an original study of MR [26], we presented two line drawings of three dimensional (3D) objects (target images) simultaneously on the screen. Each object subtended 9.4 degrees in visual angle and consisted of eleven cubes attached face-to-face to form an arm-like structure with three elbows. One object was presented at a position right to a fixation point while the other appeared in the left visual field of subjects. A center-to-center distance between the two objects was 11.7 degrees. In a half of trials, the two drawings portrayed an object with the same 3D structure but from different viewpoints (same trials). An angular difference between the two images was varied across trials from 0 to 180 degrees in a step of 20 degrees. In the other half, the drawings depicted two different 3D shapes, with one object being a mirror-reversed image of another (different trials). The angular difference between the two objects also randomly changed from 0 to 180 degrees as the same trials. Subjects judged whether the two objects shared the same 3D structure or not as quickly and accurately as possible. They pressed one key when the two objects were congruent with respect to 3D shape (“same” response) and pressed another when not (“different” response).

Each trial began with a fixation for 500 ms, followed by the target images of 3D objects. The targets remained on the screen until subjects pressed any key. After a practice session of 20 trials, they completed 4 main sessions of 60 trials, resulting in 240 trials in total. An order of 20 types of trials (same/different × ten rotation angles from 0 to 180 degrees) was randomly intermixed within each session.

**Figure 1:**
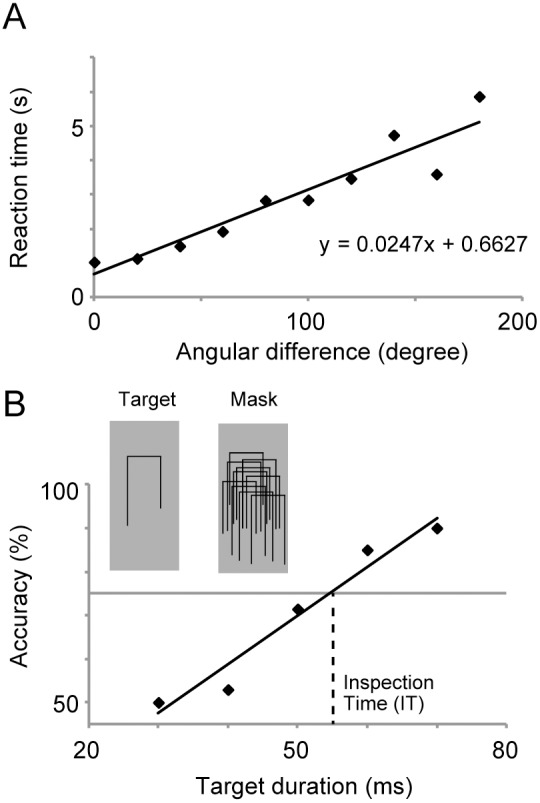
Example stimuli and data analyses in mental rotation (MR) and inspection time (IT) tasks. In the MR task (panel A), subjects were instructed to answer whether two images of three-dimensional (3D) objects (each consisted of eleven cubes attached face-to-face, see Methods for details) were the same or different in their 3D structures. We plotted reaction times as a function of angular differences between the two shapes and estimated a slope and intercept with a linear fitting. The smaller slope of the linear function represents a higher speed of mental rotation. In the IT task (panel B), target and mask stimuli were successively presented near a fixation point. Subjects judged whether the left or right vertical line of the target was longer. In the figure above, a correct answer is left. A stimulus onset asynchrony (SOA) between the target and mask started from 80 ms, being changed in a step of 10 ms based on an accuracy of the last two trials. We plotted the accuracy as a function of SOA, estimating a 75% threshold with a linear fitting as an index of the IT. The smaller IT represents a faster speed of object recognition.

We analyzed the data of the same trials when subjects answered correctly. For each subject, changes in reaction times were plotted as a function of angular difference between the two objects (**Fig. 1A**). We applied a linear regression to those data and estimated a slope α of a fitted line as an index for a speed of mental rotation [27]. A smaller α represents a faster speed of rotation. Median and inter-quartile range of a determination coefficient of linear fittings (*r* ^2^) were 0.313 and 0.195–0.398, respectively. The responses in the different trials were excluded from these analyses because a previous study has reported a departure from linearity of those data [28]. Indeed, our present data showed a determination coefficient significantly lower in different than same trials (*t*(45)  = 12.6, *p*<0.001).

#### Inspection Time (IT) task

Procedures of the IT task conformed to those of a previous study [29]. A target stimulus (‘pi-figure’) consisted of a pair of vertical parallel lines connected to a horizontal line at their top (inset of **Fig. 1B**). In a random half of trials, the left vertical line was slightly longer than the right while the right vertical line was longer in the other half of trials. The line lengths for the target-stimulus were 3.4, 5.1, and 6.8 degrees for the horizontal, short-vertical, and long-vertical lines, respectively. Immediately following a presentation of the target, a mask (duration: 300 ms) was presented to disrupt the processing of an iconic image of the target. The mask was composed of ten pi-figures randomly placed at neighboring areas of the target. Subjects were instructed to indicate whether the left or right vertical line was longer by pressing one of two keys. Following a participant’s response, the next trial began. Although the target was presented for 80 ms in initial two trials, we varied the duration of the target stimulus in every two trials based on the task accuracy. The duration was decreased by 10 ms if a subject correctly answered in both of the last two trials, whereas it was increased by 10 ms if a subject responded incorrectly to one or both trials. Subjects completed 3 sessions of 60 trials after 20 practice trials.

For data analysis, we investigated changes of the accuracy in each duration of the target. When a number of trials in a given duration was 10 or less, those data were excluded from the analyses. As an index of the inspection time, we estimated the 75% threshold of those changes in task accuracy [29] using a linear interpolation (**Fig. 1B**).

#### Raven’s advanced progressive matrices test

General fluid intelligence of subjects was measured as the number of correct answers on 36 items of Raven’s Advanced Progressive Matrices test (APM) [30]. We chose the APM because it had been widely used in previous studies on a relationship between mental speed and intelligence [7], [10]. Our use of the APM in the present study thus would facilitate a comparison of results between previous and present studies. In APM, each item was composed of a grid of eight black and white figures placed in a 3×3 matrix with one blank. The figures ranged from geometric shapes to textured patterns. Subjects were instructed to select one figure (out of eight figures below the matrix) that would best fill the blank. The 36 items were arranged in an order of increasing difficulty. Responses were made using paper and pencil. We administered the APM with a time limitation of 20 minutes. A previous study has shown that this 20-minute timed version could be used as an adequate predictor of the untimed APM scores [31].

### Correlation Analyses

We analyzed correlations of behavioral measures in the MR and IT tasks with the APM scores of the same group of subjects. Those correlation analyses were performed with Matlab robust correlation toolbox [32]. First, we estimated a center of the multivariate data with minimum covariance determinant estimator [33], [34] by calling the LIBRA toolbox [35]. Bivariate outliers were then identified using a projection technique based on the box-plot rule [36], [37]. Finally, we computed Peason’s correlations with the outliers excluded from the computation. No outlier was actually identified in our main analyses (IT vs. APM, MR slope vs. APM, MR intercept vs. APM, etc.).

## Results

### Correlation of Mental Speed of Rotation with Fluid Intelligence

In the MR task we estimated three measures for each subject; an overall accuracy, a slope and an intercept of reaction times as a function of angular differences. Mean (±SE) across 46 subjects were 93.3±0.84% (accuracy), 0.021±0.003 s/deg (slope), and 1.037±0.098 s (intercept), respectively. On the other hand, mean (±SE) score of Raven’s APM was 25.72±0.43. When we investigated the relationship of those APM scores with the three MR measures, a significant correlation was found between the APM scores and MR slope (*r* = −0.292, *p* = 0.049, **Table 1**). Correlations between the APM and MR accuracy (*r* = 0.190, *p* = 0.21) and between the APM and MR intercept (*r* = 0.067, *p* = 0.66) were not significant. We further performed direct comparisons of magnitudes of those correlation coefficients using the Meng-Rosenthal-Rubin method [38]. The magnitude of a correlation between the APM and MR slope (0.292), however, was not significantly larger than that of a correlation between the APM and MR accuracy (0.190, *z* = 0.47, *p* = 0.64) or a correlation between the APM and MR intercept (0.067, *z* = 1.36, *p* = 017). Those results overall indicate that, of three measures in the MR task, the speed of mental rotation (slope) was significantly correlated with fluid intelligence (APM scores). Subjects with a higher score of the APM rotated mental images more rapidly.

**Table 1:** A matrix of correlation coefficients among the APM score, inspection time (IT), and behavioral measures for mental rotation (MR) task.

	APM score	IT	MR slope	MR intercept	MR accuracy
APM score					
IT	−0.341*				
MR slope	−0.292*	0.209			
MR intercept	0.067	0.055	−0.564*		
MR accuracy	0.190	−0.128	−0.115	0.031	

*p<0.05.

### Correlation of Inspection Time with Fluid Intelligence

Mean (± SE) inspection time (IT) were 49.7±2.0 ms. When those ITs were compared with the AMP scores, we found a significant correlation between these two (*r* = −0.341, *p* = 0.02). Those results replicated the previous findings [10] and showed that subjects with higher score of the APM recognized a shape of the target more rapidly.

### Correlation of Mental Speed of Rotation with Inspection Time

We finally analyzed the correlation between the three measures of MR and IT. No significant correlation was observed between the MR slope and IT (*r* = 0.209, *p* = 0.16), between the MR intercept and IT (*r* = 0.055, *p* = 0.72), or between MR accuracy and IT (*r* = −0.128, *p* = 0.40). Since the MR slope and IT were individually correlated with the APM (see above), we also computed partial correlation coefficients between the MR measures and IT controlling for the APM. Those partial correlations were *r* = 0.121 (*p* = 0.43) for the MR slope vs. IT, *r* = 0.083 (*p* = 0.59) for the MR intercept vs. IT, and *r* = −0.068 (*p* = 0.66) for the MR accuracy vs. IT. There was thus no relationship between the performance of the MR task and that of IT task.

## Discussion

In the present study, we simultaneously investigated the relationship among three psychological measures: APM (an index of fluid intelligence), IT, and MR. Correlation analyses on those measures resulted in statistical tests for 10 times in total (**Table 1**). In addition to a significant correlation between the IT and APM scores previously reported, we found that the slope of MR was significantly correlated with the APM. In contrast, no correlation was found between IT and any MR measures. Our results overall indicated two types of speed measures in visual processing (MR slope and IT) independently correlated with fluid intelligence (APM scores).

Several studies have investigated correlations between MR and an index for fluid intelligence (e.g. IQ) [28], [39]–[41]. Those results were intermixed. Some studies found a significant correlation of intelligence with MR measures [39], [40], while others did not [41]. Another study showed that MR measures were significantly correlated with intelligence when task stimuli were unfamiliar to subjects although they were not correlated when the stimuli were familiar [28]. These confusing results could be partly attributed to the difference of the MR measures. Some studies investigated the correlation of intelligence with the overall accuracy or RT of MR, while others calculated the slope of RT. Because the MR task includes so many mental processes (e.g. visual recognition, manipulation and comparison of mental images, and perceptual decision making) [39], recent studies analyzed the data of MR using separate measures (e.g. intercept, slope). The slope of the RT increase as a function of rotation angles is now assumed to be the most appropriate index for the speed of mental rotation [27]. The intercept of that function, on the other hand, reflects general speed of task performance (visual recognition, decision making and an execution of motor responses). In our study, we individually compared those two indices (slope and intercept) with APM scores, finding a significant correlation between the slope (but not intercept) and APM. Our data thus presented stronger evidence for the relationship between the rotation speed and fluid intelligence.

**Figure 2:**
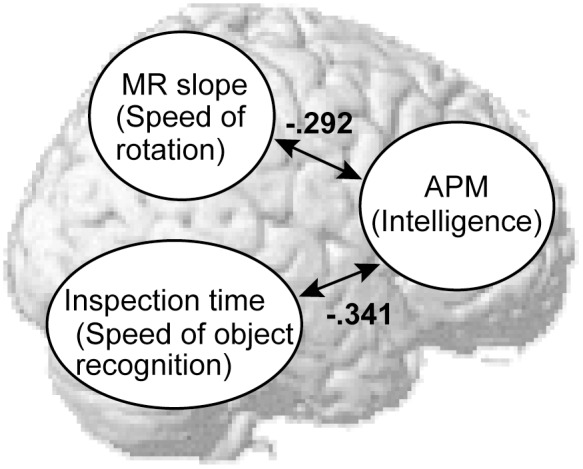
The relationship among three psychological measures (APM scores, MR slope, and IT) depicted over a template brain image in Statistical Parametric Mapping (SPM, available online athttp://www.fil.ion.ucl.ac.uk/spm/software/spm8/). Previous studies have indicated a close relationship between intelligence and a function of the frontal cortex. A speed of object recognition (IT) reflects a function of the ventral pathway (from the occipital to temporal regions), while the rotation of mental images is mainly performed in the dorsal pathway (from the occipital to parietal regions). Our study found that the MR slope (not intercept) and the IT were individually correlated with the APM scores, suggesting that the speed of mental rotation and object recognition reflect different factors of fluid intelligence.

Another feature of our study is that, in addition to the MR and APM scores, we also measured the IT of the same subjects as an index for a speed of object recognition. Consistent with previous studies, we found a significant correlation between the IT and APM [10]. In contrast, none of the MR indices (slope, intercept, and accuracy) correlated with the IT significantly, which indicates the independency of the two measures. Our result thus suggests a model in **Fig. 2** where mental abilities related to the two perceptual tasks (IT and MR) differently contribute to fluid intelligence. This view is consistent with the two-pathway model of visual processing in neuroscience [11]. It has been suggested that a performance of object recognition task (e.g. IT) reflects a function of the ventral pathway [14], while the rotation of mental images is mainly processed in the dorsal pathway [19], [20], [22]. Although previous studies focused on the relationship between intelligence and neural processing in the frontal cortex [42], our current results showed that intelligence is also supported by two fundamental cognitive functions mutually independent, one based on the ventral and another based on the dorsal pathways in the human brain.

The present study has some implications for the mental speed hypothesis previously proposed [3], [7]. This hypothesis assumes a universal factor for a speed of mental processing; a smarter brain processes information faster in any visuo-cognitive tasks. In the current study, strong correlations were found between the rotation speed (MR slope) and APM, and between the recognition speed (IT) and APM. Those strong correlations indicate that fluid intelligence is closely related to the speed of various cognitive tasks, which supports the mental speed hypothesis. A notable thing was, however, no direct correlation was found between the IT and MR. This lack of correlation means that there are at least two types of “speed” in mental processing, one related to objects recognition (IT) and another related to manipulations of mental representations (MR). Our results thus suggest a new possibility that those two types of mental speed differently contribute to intelligence.
